# Family members’ experiences of palliative sedation -a systematic integrative review

**DOI:** 10.1186/s12904-026-02025-z

**Published:** 2026-02-19

**Authors:** Linda Bäcklund, Marie Widegren, Åsa Rejnö

**Affiliations:** 1 The Palliative Consultant Team Falkenberg, Hallands Hospital Varberg, Region Halland, Varberg, Sweden; 2 The Palliative Team, Hospital in the West, Alingsås Hospital, Alingsås, Sweden; 3 Department of Health Sciences, University West, Trollhättan, Sweden; 4 Skaraborg Institute of Research and Development, Skövde, Sweden; 5 Department of Medicine, Skaraborg Hospital, Skövde, Sweden

**Keywords:** Experiences, Family members, Feelings, Palliative sedation, Terminal sedation

## Abstract

**Background:**

Palliative care means care in the event of an incurable illness where the intention is to alleviate suffering and promote quality of life. In cases where the symptoms cannot be fully alleviated, palliative sedation may be required which is a lowering of consciousness in patients using medication to alleviate their suffering.

**Aim:**

The aim of this systematic integrative literature review was to describe how palliative sedation for patients at the end of life is experienced by their family members.

**Method:**

A systematic integrative review as described by Whittemore and Knafl was performed using three databases; CINAHL, PubMed and APA PsychInfo. Eleven peer-reviewed studies published between 2005 and 2025 met the inclusion criteria. They described family members’ firsthand experiences with ethical considerations clearly stated and were not excluded for including participants under 18, being review articles, or being published in a language other than English.

**Results:**

In total, the eleven studies comprised 1061 family members. Three of the studies came from Japan, two from the Netherlands, two more from the Netherlands, Belgium and UK and one from Belgium, Israel, Switzerland and Taiwan, respectively. Seven studies used quantitative methods, and four used qualitative methods.

The results demonstrate how family members of a seriously ill person experience palliative sedation and are organized into eight categories: *Affects the lifespan, Creates anxiety, Emphasizes importance of communication, Facilitates a good death, Highlights significance of participation, Impacts time and timing, Provides a chance for farewell* and* Relieves suffering.*

It was found that many family members of patients who received palliative sedation suffered. The suffering worsened the longer the sedation lasted. Palliative sedation also provided possibilities for a good death and farewell even though it was found to affect the life span. Clear information about what to expect and a good relationship with the healthcare staff was crucial.

**Conclusion:**

The well-being of family members was affected while the patients received palliative sedation, and they felt worse the longer the sedation lasted. Consistent information from the healthcare staff was of great importance. The lack of international guidelines and registries contributes to ambiguity regarding the definition and prevalence of palliative sedation in published articles, thereby limiting the scope of comparative research. Nevertheless, palliative sedation constitutes an important component of palliative care, underscoring the need for further studies, including examinations of cultural and religious influences on family members’ experiences.

**Supplementary Information:**

The online version contains supplementary material available at 10.1186/s12904-026-02025-z.

## Background

Palliative sedation is an effective relief for severe symptoms at the end of life, involving the reduction of the patient's consciousness to varying degrees with the help of medications. In modern society, there are expectations that dying should be without suffering, and therefore, patients and their families increasingly view palliative sedation as a right to die in their sleep [1]. In countries such as the Netherlands and Switzerland, but also Sweden, there has been a clear increase in palliative sedation between 2005 and 2020, and all signs suggest that it is also increasing in other countries worldwide [2, 3]. There are also indications that it is not solely limited to cases involving refractory symptoms [4]. Reasons for the rise in palliative sedation is believed to be an increased awareness among patients and families that this treatment option exists, greater knowledge among healthcare staff, and the use of palliative sedation for non-physical symptoms such as fear and anxiety [2]. Care during the final stage of life is often referred to as palliative care. The World Health organisation (WHO) describes palliative care as a human right that should be accessible to all populations [5]. According to Worldwide Hospice Palliative Care Alliance (WHPCA) [6] the intention is to relieve suffering and promote quality of life for both patients and their family members. In recent years, another definition has gained traction in the global perspective. International Association for Hospice and Palliative Care (IAHPC) [7] defines palliative care as a holistic approach to individuals of all ages experiencing serious suffering. The intention is to provide support to patients and their families based on their individual needs throughout the illness process while respecting their cultural values. Death is seen as a natural process that is neither hastened nor delayed [7]. However, global access to palliative care varies widely, and despite increasing provision, only about 12% of those in need have their needs fully met [6]. Although most people in need of palliative care live in low- and middle-income countries [5], approximately 70% of palliative care services are concentrated in high-income countries [8]. In countries where palliative care is sparsely implemented or not implemented, the likelihood that palliative sedation is available is very small.

### Palliative sedation – definition, principles and guiding frameworks

Even with well implemented palliative care and effective symptom management, patients may still experience severe symptoms at the end-of-life [9]. In such cases, palliative sedation may be considered as a treatment option that can be carried out both in healthcare facilities and in the patient’s home. Palliative sedation is defined in various ways, with ‘*reduced consciousness*’ being the only consistent element across definitions [10]. We use the definition for palliative sedation given by IAHPC “*The monitored use of medications intended to induce a state of decreased or absent awareness (unconsciousness) in order to relieve the burden of otherwise intractable suffering in a manner that is ethically acceptable to the patient, family, and health care providers*” [11]. Palliative sedation is differentiated from both euthanasia and assisted dying (including physician-assisted suicide and physician-assisted dying) by its lack of intent to end a person’s life. Palliative sedation is provided to alleviate refractory suffering from severe physical, psychological, or existential distress at the end of life when conventional treatments fail to provide relief [12]. Currently there are no globally established guidelines for palliative sedation. However, a recommended framework has been developed by the European Association for Palliative Care (EAPC) based on input from experts in 28 countries foremost from Europe, with representation from researchers in Algeria, Canada, Israel, Japan, and the United States of America [12]. There is general consensus regarding the principle of proportionality in palliative sedation, which applies to two key dimensions: the depth of sedation and its timing. For palliative sedation to be initiated, a clear medical indication must be present, and the treatment should be proportional to the severity of the symptoms [12]. The lowest dose of sedative medications that effectively relieves suffering while still allowing the patient to interact as much as possible with their surroundings is sought. For initiation, the patient’s experience of the symptoms and their severity, should be given significant consideration. Additionally, the patient should be involved in decisions regarding available treatment options. Furthermore, consultation with the entire care team involved is essential, as is addressing the needs and concerns of family members. Palliative sedation can range in depth from mild to deep and may be administered intermittently, via scheduled injections or continuously, often using a pump. The principle of proportionality is considered most favourable, concerning both depth of sedation and timing. Intermittent sedation may be appropriate earlier in the illness trajectory to provide temporary relief while other treatments are adjusted and to offer a respite from suffering, while continuous sedation is generally reserved for the final phase. It is also recommended, when feasible, to pause sedation periodically in order to allow for reassessment of the patient's condition [12]. The EAPC guidelines also note that experts were unable to reach a consensus on a precise definition of the end-of-life phase, making it difficult to establish a universally applicable time frame for when continuous deep sedation (CDS) should be initiated.

The prevalence of palliative sedation is strongly influenced by organisational, legal, and cultural contexts and closely reflects the overall development of palliative care within a country, being rarely offered in less developed care systems and more frequently provided where palliative care is well established [12, 13]. Globally, palliative care is classified into six levels of development. The highest level is characterised by full integration into healthcare systems, unrestricted access to strong opioids, and academic-level education, while the lowest level involves no known provision of palliative care [14] and consequently no provision of palliative sedation. There is no comprehensive global overview of neither the prevalence of palliative care nor the availability of palliative sedation. It is estimated that 10–18% of all deaths among patients with palliative care needs in Europe have received palliative sedation [12]. To gain a sense of the extent, we have compiled data from several sources to attempt to provide as good global overview as possible (Table 1).

**Table 1:** Palliative care availability, integration, contexts, and sedation practices by continent

Continent	Palliative care availability	Level of integration*	Context for palliative care	Prerequisites for and occurrence of palliative sedation
Asia [15]	Poorly implemented, low awareness, incomplete and inadequate care in most countries	1 (Japan) −6 (Laos)	Available in several care contexts, great variation between the countries	Accessibility and availability of opioids are limited, palliative sedation is accordingly scarce
Africa [16]	Poorly implemented, absent or scarce in many countries	1 (Malawi) – 6 (several countries in Central Africa)	Mostly in home-based care	Many countries restrict prescription of opioids; palliative sedation is accordingly scarce
Australia/Oceania [17, 18]	Well implemented but shortage in staff exist	1 (Australia and Oceania)	Available in most care contexts	Palliative sedation is part of the palliative care but not fully implemented in rural settings and low-resource environments
Europe [12]	Well implemented in majority of countries	1 (majority of countries) – 6 (Montenegro)	Available in several care contexts, variation across countries	Generally good access to opioids, palliative sedation implemented in countries with high level of integration of palliative care
Latin America [19, 20]	Implementation varies across countries, ranging from isolated service delivery to advanced integration within standard healthcare systems	1–6, highly varied	Foremost home care teams and hospital support services	Access to opioids differ between countries from scarce to very good, unclear if palliative sedation is implemented
North America [21–23]	Well implemented and accessible	1	Exist in several care contexts	Framework is in place (Canada), National palliative care register (US), opioids are available, palliative sedation is implemented

Availability of palliative care, level of integration of palliative care, contexts for palliative care, prerequisites for and occurrence of palliative sedation given by continents of the world

^*^As described by D Clark, N Baur, D Clelland, E Garralda, J López-Fidalgo, S Connor and C Centeno [14]

### Family members of patients at end-of-life

It has long been known that those around patients in the final stages of life are in a vulnerable situation, one that is most often entirely new to them. These individuals can be defined in different ways, and in this study, we use the concept “family member” referring to a person whom the patient considers having a close relationship with. One key factor for family members to feel secure during this time is reassurance that the patient is living as well as possible, despite the impending approach of death [24, 25]. It is also common for family members to express need for care for the ill person rather than own needs [26]. This challenging situation can manifest as anxiety and depression and diminished health among the family members [25, 26]. Therefore, it is crucial that family members' needs for help and support are addressed. In a study on medical records of deceased patients it was concluded that family’s needs had been assessed for only 52% of the patients, with a higher chance for this if family members were under the age of 65 [27].

The main finding from systematic reviews [28–30] and empirical studies [26, 27, 31, 32], conducted in both developed and developing countries, is that families in general have a significant need for information when patients are approaching end of life. Family members in developing countries to a higher degree had needs related to costs for medication and care for the dying person [29]. The studies, conducted in rural areas [29], hospital settings [26], hospices [31] and home care settings [27, 30, 32, 33] all emphasize the need for information. This includes among other things, information on prognosis, what to expect during the final phase of life, visible changes in the patient’s appearance to expect near death and how to best care for the dying person. The need for emotional and psychological support also was shown [28, 29, 31, 33].

Family members’ need for information is just as critical when the question of palliative sedation comes into play. A previous review indicates that family members of patients who received palliative sedation reported a substantial distress in connection with it [34]. The need for improvement in the information given was noted. However, that review includes not only studies with first-hand information from family members but also those based on data from medical records and information provided by proxies, such as physicians and nurses. One such example is a study from the Netherlands [35] based on notations in medical records by the multidisciplinary teams. That study found that if family members perceived the patient as suffering, it also led to suffering within the family members including issues such as sleep deprivation, exhaustion and anxiety. About one third of the family members also reported suffering due to concerns that the patient was suffering during the sedation. Moaning, anxiety, and restlessness in the patient were expressions given as examples of what the family members could interpret as suffering. The medical records also indicated that the longer the palliative sedation lasted, the greater the strain and worry on the family members.

Since palliative sedation is initiated to alleviate refractory suffering from severe physical, psychological, or existential distress, the illness trajectory leading up to its initiation is often stressful for family members as well. Although its clinical use is increasing, knowledge and understanding of palliative sedation remain limited and, at times, ambiguous among healthcare professionals, family members, and the general population. In the public discourse, palliative sedation is frequently conflated with euthanasia [36] and studies indicate that nurses may question whether palliative sedation contributes to patient death and perceive it as closely related to euthanasia [37, 38]. Such uncertainties risk shaping how palliative sedation is communicated, perceived, and experienced by family members. At the same time, the use of palliative sedation has increased in recent years, while research specifically addressing family members' experiences remains scarce and, in some cases, relies on second-hand accounts rather than first-hand perspectives [34]. Although family members of critically ill patients receiving palliative sedation are known to face profound emotional and ethical challenges, empirical knowledge about how they experience and make sense of these situations remains fragmented.

Moreover, studies focusing on healthcare professionals suggest that situations in which palliative sedation becomes relevant are often perceived as particularly distressing for family members, further underscoring the need to better understand their experiences. Taken together, these gaps in the literature highlight the need for a comprehensive and up-to-date synthesis of existing research on family members’ firsthand experiences of palliative sedation. Such knowledge is essential to inform clinical practice, improve communication and support, and ultimately promote family members’ well-being in line with the Sustainable Development Goal 3 (SDG) [39].

### Aim

The aim of this systematic integrative literature review was to describe how palliative sedation for patients at the end of life is experienced by their family members.

## Method

### Design

An integrative design with an inductive approach was the starting point for this literature-based study. The integrative method was chosen as it allows for the inclusion of studies conducted using diverse methodologies to understand a phenomenon or problem [40]. For this type of review, it is emphasized that extra clarity regarding the aim is required, as the integrative review can include a variety of data, and it is important to extract the right data. Although integrative review as a method can use various sources of information [41], we chose to include only peer-reviewed articles with different research designs. The study followed the steps by Whittemore and Knafl, problem identification, literature search, data evaluation, data analysis and presentation [40] and is reported in accordance with Preferred Reporting Items for Systematic Reviews and Meta-Analyses (PRISMA) the co-decide systematic integrative review, adapted from PRISMA [42].

### Problem identification and literature searches

The problem identified in this review is exploring how palliative sedation for patients at the end of life is experienced by their family members, according to Whittemore and Knafl integrative approach [40].

The PEO (Population, Exposure, Outcome) framework was applied to the research question to divide it into its elements to enable a structured search and define inclusion and exclusion criteria. The PEO structure and the application of it to the study aim is described in Table 2.

**Table 2:** PEO framework and the study aim

P- Population	E- Exposure	O- Outcome
Family members of patients over 18 years old who are affected by a serious illness and have a palliative care need where palliative sedation is relevant	Palliative care Palliative sedation	Family members experiences

To get a grip of the problem, broad searches were initially performed to find relevant search terms. The authors conducted numerous searches and tested a variety of keyword combinations before settling on the final search strategy. No subject terms or MESH-headings were used, as there were none relevant to our search terms in the databases used. The search terms were adjusted for respective database and new search terms were generated through the different search functions in the databases used. During this process, the authors also sought assistance from a librarian to ensure that our search strategy was optimized and that we would not risk missing relevant articles. The Boolean operators AND and OR were used to form blocks, which were combined, and truncation were used to broaden the search and increase the number of articles found when appropriate. The words finally used in the searches were: continuous sedation, deep sedation, palliative sedation, terminal sedation, attitude, experience, view, feeling, perspective, “family attitude”, “life experience”, family, “loved one”, next of kin, partner, relatives, spouse, “significant other”, see supplementary file 1 for details on search strategy.

The search was initially conducted in January 2023 and remade in July 2025. Systematic searches were performed in Cumulative index of nursing and allied health (CINAHL), Public/Publishers Medline (PubMed) and American Psychological Association (APA PsycInfo). Articles published from 2005 were sought. The wide time frame was applied since research on the topic is scarce and there has been a substantial development in the area during more recent years and from year 2005 the research took off.

The inclusion criteria were peer-reviewed original studies regardless of method used, describing family members’ firsthand experiences of palliative sedation, and with explicitly stated ethical considerations. Exclusion criteria were articles concerning participants under age 18 as the person receiving palliative sedation, review articles and studies in languages other than English. Palliative sedation for children is very rare and presents additional challenges for family members, not directly related to the sedation itself, such as the loss of a child, and was therefore excluded. No limitations according to geographical locations were set.

**Fig. 1:**
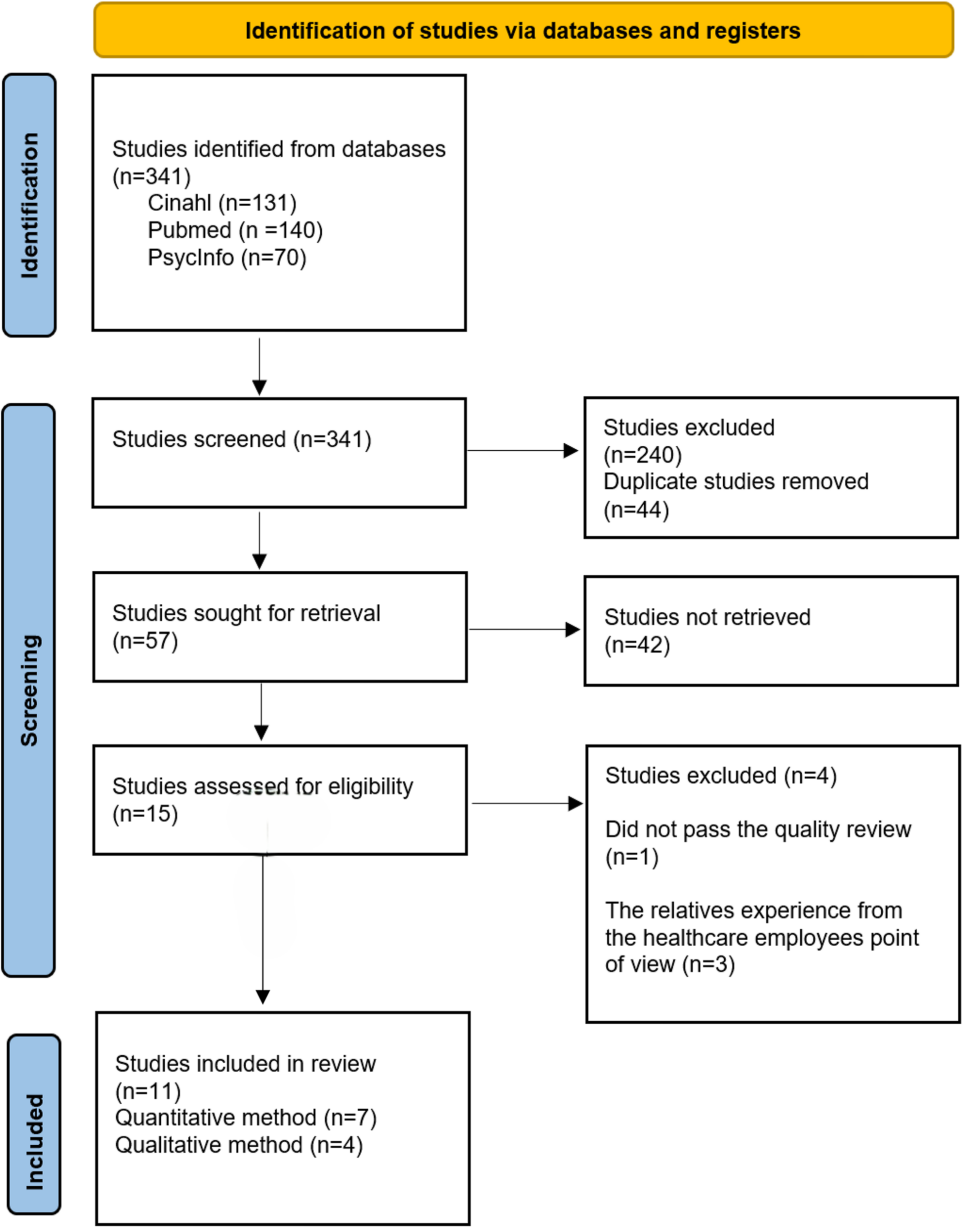
PRISMA flowchart about here

From the total of 341 articles identified through the searches, firstly duplicates were removed. After that titles and abstract were independently assessed by the first two authors, whether to include for screening of the articles. All articles included for screening in full text were read and independently assessed by the first two authors and thereafter in the research group as a whole, for consensus decisions on which articles to include in the review. The process of inclusion and exclusion is reported according to the PRISMA structure (Preferred Reporting Items for Systematic Reviews and Meta-Analyses) [43], see Fig. 1.

### Data evaluation

Data was evaluated through a review of the studies’ quality in accordance with what is suggested by Whittemore and Knafl [40]. For the review, the NICE quality assessment tools (National Institute for Health and Care Excellence) were used for both qualitative and quantitative methodologies [44, 45], as these could be applied to articles with various research designs. All articles but one were found to be of high quality, including low risk of bias, and were included in the study.

### Data analysis

Great attention was given to the analysis to ensure it was as accurate and comprehensive as possible, and the results were discussed within the entire research group. The first step in the analysis is data reduction, according to Whittemore and Knafl [40]. Based on the selected articles, key findings were identified that described how palliative sedation for seriously ill individuals was experienced by their family members, which was reflected in 15 articles. The reference lists of the articles were reviewed, but no further articles were identified through this process. Four articles were excluded: one did not pass the quality assessment, and three were excluded since they were based on family members' experiences as interpreted through the staff’s notes in medical records and interviews with staff. Ultimately, 11 articles were used for the results.

*Data overview *is the second step during which data from different articles are compared, resulting in the emergence of various themes [40]. The articles were read multiple times by the authors LB and MW to gain a comprehensive understanding of the content. Results that aligned with the purpose of the literature review were then extracted.

In the third step, *data comparison*, different patterns, relationships, and ultimately various themes are identified [40]. The key findings from all the articles were read multiple times by LB and MW, both individually and together, until differences and similarities became evident. These differences and similarities were used to create categories. The categories were then reviewed and tested repeatedly, both separately and collectively, within the research group as a whole. Ultimately, eight main categories emerged.

*Conclusion and verification* are the final step in the analysis [40]. Based on the eight main categories, conclusions were drawn regarding how palliative sedation for seriously ill individuals was experienced by their family members, which constitutes the results.

## Results

### Characteristics of the studies

This integrative literature review included eleven empirical studies representing the same number of primary studies published between 2009 and 2022. Three of the studies came from Japan, two from the Netherlands, two more from the Netherlands, Belgium and UK and one from Belgium, Israel, Switzerland and Taiwan, respectively. The research designs were qualitative (*n* = 4) and quantitative (*n* = 7). The qualitative studies included a total of 101 family members, with data primarily collected through individual interviews, though some also used focus group interviews and questionnaires. The quantitative studies involved 960 family members, all utilizing questionnaires for data collection. The care contexts of the studies included ordinary housing, nursing homes, long term institutions (mainly for elderly people), palliative and specialised palliative care units, hospitals (mostly oncology wards, intensive care units and hospices). The diagnoses of the patients were predominantly cancer [46–52], with mixed diagnosis such as cancer, Alzheimer’s disease, dementia and ageing [53], cancer, Amyotrophic lateral sclerosis (ALS), lung fibrosis and Friedreich’s ataxia [54]. Diagnoses were not stated for two studies [55, 56]. See Table 3 for details on the included studies.

**Table 3:** Characteristics of included studies

Author, year, reference and country	Aim	Method	Sample	Type of sedation	Main Findings
Bruinsma et al., 2013 [53] The Netherlands	To explore relatives’ experiences with palliative sedation and to gain more insight in positive and negative elements in their evaluation of palliative sedation	Qualitative method, focus groups and individual interviews Analysed using constant comparative analysis	14 relatives of patients who received palliative sedation until death in various care settings in the Netherlands	Not stated	Palliative sedation was initiated with the aim of relieving symptoms. Relatives felt that death occurred naturally and without struggle, which was a relief, although the burden on them increased as the sedation continued. They expressed that it was difficult to stand by and reported dissatisfaction with not having received enough information. Sedation provided an opportunity for saying goodbye
Bruinsma et al., 2014 [46] The Netherlands, Belgium and the United Kingdom	To explore relatives’ descriptions and experiences of continuous sedation in end-of-life care for cancer patients and to identify and explain differences between respondents from the Netherlands, Belgium, and the UK	Qualitative method, focus groups and in-depth interviews. Qualitative analysis software (NVIVO 9) was used to organize the data	In-depth interviews conducted between January 2011 and May 2012 with 38 relatives of 32 cancer patients who received continuous sedation until death in hospitals, community settings, and hospice/palliative care units in the Netherlands, Belgium, and the United Kingdom. A total of 84 deceased patients were included as case studies	Continuous	Relatives believed that the sedation contributed to a good death by providing effective symptom relief and allowing the patient to find peace and die with dignity. Relatives expressed increased concern when the sedation was prolonged. In Belgium and the Netherlands, there was a stronger perception of having received adequate information, and sedation was not seen as hastening death—unlike in the United Kingdom
Bruinsma et al. 2016 [47] The Netherlands	To examine whether the relatives of patients who receive palliative sedation differ in their experience of the dying phase and their wellbeing after the patient’s death compared to the relatives of patients who died a non-sudden death without the use of palliative sedation	Quantitative method, observational survey study with questionnaire. Multivariable linear regression analysis and logistic regression	Relatives of 564 patients who died a non-sudden death at Hospice 'Laurens Cadenza' or the Erasmus MC Cancer Institute in Rotterdam between 2010 and 2013. Of these, 243 relatives were willing to participate in the study	Not stated	Palliative sedation has no negative impact on relatives' experience of the patient’s final days or on the relatives’ well-being after the death. Relatives felt they had been informed about the patient’s condition and had been given time to say goodbye
Imai et al., 2022 [48] Japan	To compare family experience between families of patients who received proportional or continuous deep sedation (CDS)	Quantitative method, multicentre prospective observational study supplemented by an anonymous, cross sectional, self-reported Questionnaire survey. Descriptive analysis and Mann–Whitney U test or chi-squared test (Fisher’s exact methods). Effect sizes (ES) were calculated using Cohen’s d	Questionnaires sent to relatives in October 2020, with responses received from 78 relatives (intermittent sedation: 58, continuous sedation: 20) Cohort 1: relatives of patients who participated in a prospective multicentre observational study. The patients were adults with cancer treated at 23 specialized palliative care units in Japan Cohort 2: relatives of patients who died between April 2018 and March 2020 at Seirei Hospice in Japan	Proportional sedation or continuous deep sedation	Relatives were satisfied with the palliative sedation, which was initiated at the right time and relieved symptoms, but in one-third of the cases, they experienced distress and felt concerned. The time until death was perceived as prolonged, and there were worries about legal issues. Relatives felt supported by the healthcare staff
Koike et al., 2015 [49] Japan	- To explore the efficacy of an MDTC (Multidisciplinary Team Conference) concerning decision-making surrounding application for CDS until death - To understand the satisfaction and emotional distress of bereaved families related to CDS	Quantitative method, medical and nursing records, questionnaire Statistical analysis	Relatives of 1,581 cancer patients who died in a palliative care unit at Higashi Sapporo Hospital in Japan between April 2005 and August 2011 Relatives of the 22 patients who received palliative sedation were given a questionnaire, 13 responded to the questionnaire	Continuous deep sedation	Relatives were satisfied with the palliative sedation, though half of the families experienced emotional distress due to the sedation. Family members expressed concern about being unable to communicate with the patient and about the possibility that the sedation might shorten the patient's life
Morita et al., 2019 [50] Japan	To make rough comparisons of opinions about CDS among patients with cancer, bereaved families, home physicians, and palliative care specialists	Quantitative method, Questionnaire Descriptive statistics, analysis of variance and Tulsky method as a post hoc test	Patients were identified via a database Four surveys were sent between August 2016 and February 2018 to patients, one family member per deceased patient who died in one of 71 inpatient or palliative care units, general practitioners, and palliative care specialists. The sample included 412 patients, 512 bereaved family members, 131 general practitioners, and 440 palliative care specialists	Continuous deep sedation	Patients and their families considered continuous deep sedation appropriate for those experiencing severe symptoms causing both psychological and physical suffering. Sedation was seen as providing a good death for the patient. Symptom relief was valued more highly than survival
Raus et al., 2014 [55] The United Kingdom, The Netherlands and Belgium	To provide insight into what may influence how professional and/or family carers cope with such distress	Qualitative method. Interview study. Constant comparative method according to Corbin and Strauss	57 physicians, 73 nurses, and 34 relatives of deceased patients in the United Kingdom, the Netherlands, and Belgium. Interviews were conducted between January 2011 and May 2012. The patient cases had received continuous palliative sedation at home, in hospitals, or in palliative care units in the UK, the Netherlands, or Belgium	Light and deep continuous sedation	Relatives found it difficult to be physically close to a patient if they perceived that the patient was suffering. They also found it hard to see the patient when severe symptoms were present and they were unable to help
Shen et al. 2018 [51] Taiwan	To examine and compare family concerns about PST (Palliative sedation therapy) use and its effect on the grief suffered by terminally ill patients’ families in palliative care units (PCUs) or intensive care units (ICUs)	Quantitative method. Observational study, questionnaire. Descriptive statistics, Chi-squared and t-tests, linear regression analysis	154 family members of patients were recruited from palliative care units and intensive care units in Taiwan. 143 completed the study, including 81 from palliative care units and 62 from intensive care units. The study was conducted in the oncology ward of one hospital and six intensive care hospitals in Taipei, Taiwan	Not stated	Relatives of patients cared for in a palliative care unit expressed concern that there might be other ways to relieve symptoms besides sedation. Relatives of patients treated in the ICU felt that more could have been done, that the sedation was not dignified, and that their level of grief was also higher
Six et al., 2020 [56] Belgium	To find out what influences professional caregivers’ and family members’ (FMs) attitudes regarding the use of monitors during CSD	Qualitative method, semi-structured interviews Grounded theory with an inductive approach according to Charmaz	15 relatives and 20 healthcare professionals who cared for patients with palliative sedation at two hospitals and two nursing homes in Belgium	Continuous sedation	Relatives considered sedation to be a good option, and monitoring equipment was seen as a way to ease their anxiety about the patient dying a painful death. They believed that the only way to ensure the patient’s well-being and that death was not being hastened was by using a monitoring system
Tursunov et al., 2016 [52] Israel	To describe the experience of family members of patients receiving palliative sedation at the initiation of treatment and after the patient has died and to compare these experiences over time	Quantitative method, questionnaire Descriptive statistics, McNemar’s test, the Test of Marginal Homogeneity, the Wilcoxon signed-rank and the paired-samples t-test Open-ended responses were thematized	Relatives of 34 patients who had been cared for at an oncology clinic in Jerusalem and received palliative sedation	Not stated	Relatives were satisfied with the palliative sedation, considered it the best way to relieve the patient’s suffering, and believed it upheld the patient’s dignity. One-third thought that the sedation shortened the patient’s life, but there were no legal or ethical concerns about the sedation
Vayne-Bossert et al., 2013 [54] Switzerland	To better know the family experience of a PS (Palliative Sedation)	Quantitative method, questionnaire Descriptive statistics, Fisher exact test and nonpaired test	An anonymous survey sent to relatives of 17 patients who received palliative sedation at the time of death, responses were received from 10 The patients were cared for in the palliative care unit at the University Hospital in Geneva	Continuous or intermittent deep sedation	Palliative sedation was administered for symptom relief and was seen as necessary by the relatives The relatives experienced emotional difficulties during the palliative sedation. Almost all of them felt they had been given the opportunity to say goodbye

*CDS* Continuous deep sedation

The results demonstrate how family members of a seriously ill person experience palliative sedation and are organized into eight categories, reported in alphabetical order: *Affects the lifespan, Creates anxiety, Emphasizes importance of communication, Facilitates a good death, Highlights significance of participation, Impacts time and timing, Provides a chance for farewell* and *Relieves suffering.*

### Affects the lifespan

Family members' perceptions of palliative sedation and its impact on lifespan varied widely ranging from beliefs that it hastened death, to views that it had no effect on lifespan, or even that it prolonged the dying process. In studies conducted in Israel, Japan, and the Netherlands some family members believed palliative sedation shortened the patient’s life [49, 52] with Tursunov et al., [52] reporting about one-third, and Koike et al. [49] about one fourth expressing this view. Some even perceived it as causing the patient’s death [52]. Despite these concerns, most family members did not believe that physicians intended to hasten death with the sedation.

Meanwhile, family members in studies from Belgium, the Netherlands, and Switzerland expressed the view that palliative sedation either did not influence the lifespan or that the possibility of it hastening death was not an issue [53–55]. Furthermore, family members of patients who received continuous palliative sedation were more likely to believe that the dying process had been unnaturally prolonged, compared to family members of patients who received proportional sedation [48]. Those who perceived the patient as being too deeply sedated were more likely to believe that death had occurred sooner than expected [51].

### Creates anxiety

Family members expressed concern for the patient's well-being during palliative sedation [46]. This concern included fears that the patient might not fall asleep quickly enough once sedation was initiated, or that they might regain consciousness during the sedation period. Some family members felt that the level of sedation was not deep enough to adequately relieve suffering [51, 55], while others perceived the patients as being too deeply sedated [51]. Concerns that alternative measures could have been used to relieve symptoms were reported in both a Japanese study [49] and a study from Taiwan [51]. In the Taiwanese study family members of patients who received palliative sedation in palliative care settings expressed more concern than those of patients sedated in intensive care units (*p* = 0.006) [51]. Some perceived that the patient was being forced to sleep and reported more intense grief than family members of non-sedated patients. Furthermore, in intensive care units where family members were not adequately prepared for the patient's deteriorating condition, there was a perception that more could have been done, and that the sedation process lacked dignity. Interviews with family members of patients who received palliative sedation in Belgium revealed anxiety and distress related to witnessing the patient's decline, changes in appearance, and also difficulty understanding how someone could survive for an extended period without food or fluids [56].

Family members described their own suffering in relation to that of the patient, highlighting the difficulty of standing by without being certain whether the patient’s symptoms were adequately relieved [53, 55]. Regardless of their attitudes toward palliative sedation, they consistently viewed the patient’s well-being as the highest priority [56]. Family members could be either satisfied or dissatisfied with the sedation itself; however, even those who expressed satisfaction still reported experiencing own emotional distress related to the patients sedation [49, 55].

### Emphasizes importance of communication

Communication with healthcare professionals about palliative sedation was uniformly emphasised as crucial by family members [46, 48, 53, 54, 56]. Clear and consistent information was considered essential, both regarding the practical aspects of initiating sedation and the overall process, as contradictory messages could lead to uncertainty and feelings of excessive responsibility [48, 53, 54]. Timely and detailed communication about what to expect, including drugs used and duration of sedation, was also regarded as meaningful [46, 47, 52]. In Taiwan, a majority of family members of patients receiving palliative sedation both in palliative care units (73%) and in intensive care units (90%), expressed a desire for the entire family to have the opportunity to discuss options for palliative sedation therapy before initiation [47]. In a study from Israel [52] and in family members from the UK [46] it was reported that families often felt insufficiently informed and wished for more comprehensive explanations, whereas most family members in Belgium and the Netherlands felt well-informed [47]. Israeli families were also often not informed about the option of palliative sedation until the patient’s condition had significantly deteriorated [52].

Cultural differences were noted in terminology: in the UK, euphemisms such as “sleeping” or “becoming calm” were commonly used, while in Belgium and the Netherlands, the term “sedation” was used explicitly [46]. A higher, though not statistically significant, proportion of family members of patients who received palliative sedation reported satisfaction with communication with healthcare professionals regarding the patient’s condition at end of life, compared to family members of patients who did not receive sedation (88% vs 82%) [47].

### Facilitates a good death

Family members stated that palliative sedation ensured a good death [56] and death with palliative sedation was also described as both 'beautiful' and 'peaceful' [46]. The fact that patients were given the opportunity to remain in a reduced state of consciousness until death, was perceived as a relief by the family members [53]. Family members of patients who received palliative sedation expressed satisfaction with the health care professionals and believed that everything possible had been done for the patient [53, 54]. Family members of patients who had received palliative sedation at the end of life rated their general satisfaction with the care at 9.4 out of 10 [54]. In addition, palliative sedation appeared to strengthen the relationship between family members and healthcare professionals, thereby enhancing their experience of the death as good. Continuous palliative sedation was associated with more time spent together with healthcare professionals, compared to family members to patients who received intermittent sedation [48].

Compared to home and palliative care physicians, family members were more likely to view palliative sedation as contributing to a good death with a pleasant care environment playing a significant role in shaping this experience and perception [50, 53]. Furthermore, more than 90% of family members reported no ethical or legal concerns related to the use of palliative sedation, nor did they feel that the patient’s dignity had been compromised [52]. Palliative sedation was seen as enabling family members to honour the patient’s wishes regarding the preferred place of death, highlighting the importance of the care setting in end-of-life experiences. Monitoring equipment in the form of an electroencephalography-based sedation monitoring system that monitors brain activity via electrodes attached to the forehead (BIS-monitor), proved helpful in assessing whether the patient’s symptoms were sufficiently relieved [57]. This reassured family members that the patient’s wish for a good death was being honoured and that suffering was minimized, which in turn supported their grieving process.

### Highlights significance of participation

Family members considered it important to be involved in decisions regarding the patient’s care and to participate in the care process. However, some family members reported that they were not sufficiently involved in the decision-making around palliative sedation [52, 53, 55]. They felt that the decision to initiate palliative sedation had been made by the physician without providing adequate information about its purpose [53]. According to the family members, it was the patient who should make the decision about sedation. Family members also reported that they had not discussed palliative sedation with the patient and that the patient had not received any explanation about the treatment [52]. When family members were not sufficiently involved in the care, they did not always feel prepared for the approaching end of life of the patient [53].

Family members’ experience of involvement did not differ significantly according to whether the patient received palliative sedation or not [47]. In the final week of life, 83% (*n* = 151) of family members of palliatively sedated patients, reported feeling involved and engaged in care compared to 74% (*n* = 90) among those whose relatives did not receive sedation.

### Impacts time and timing

Family members considered it important that palliative sedation was initiated at the appropriate time, and the duration of sedation was found to influence their overall well-being. Family members felt palliative sedation had been initiated at the right time [48, 52, 54], reported in equal proportions by family members of patients who received proportional sedation and those who received continuous deep sedation [48]. However, family members' views on the timing of palliative sedation could change over time. More family members felt that sedation had been initiated at the right time during the process than they did after the patient’s death, with some later feeling that it had been initiated too late [52]. Moreover, family members prioritised the relief of suffering over prolonged survival [50, 56].

Family members preferred that palliative sedation be limited to a short period of time, as they did not perceive a prolonged time with sedation as dignified [56]. Even though healthcare professionals had informed them that sedation could extend over a longer period, family members found it difficult to envision what such a situation would be like [46]. Extended durations of palliative sedation were experienced as burdensome, with the impact on family members increasing the longer the sedation continued [46, 53]. Nevertheless, family members considered palliative sedation to be appropriate as last resort for relief of refractory symptoms, even when it was associated with prolonged survival [50].

### Provides a chance for farewell

Family members felt that the planned initiation of palliative sedation often allowed sufficient time for farewell [46, 53, 54]. This provided both the family members and the patient with an opportunity to resolve unresolved matters and achieve a clear farewell [48]. However, not all family members viewed this experience positively; for some it felt more like a social spectacle with numerous individuals gathering to say their goodbyes, which was perceived as inappropriate [46].

A variation in how family members experienced farewell to the patient in connection to palliative sedation also showed. Family members from Belgium and the Netherlands viewed palliative sedation as an opportunity to plan a farewell, whereas this was not described by family members from the UK [46]. This difference was attributed to the fact that, in the UK, palliative sedation typically did not have a clearly defined starting point but was instead introduced gradually through the administration of increasing doses. It was also noted that if patients regained consciousness during sedation, family members in Belgium and the Netherlands found this distressing while family members in the UK welcomed it. Family members in Belgium and the Netherlands could even find it emotionally difficult to remain at the bedside after the farewell had taken place, describing the time as merely waiting for death [47].

### Relieves suffering

Family members considered the goal of the palliative sedation to be the alleviation of unbearable suffering, physically, psychologically, and existentially, and to allow the patient a dignified and peaceful death [46, 49, 54, 56]. They wished for freedom from suffering at the end of life [56] and palliative sedation was regarded as both appropriate and necessary to alleviate physical and psychological distress, including symptoms such as pain, anxiety, breathlessness, nausea, and confusion [50, 53]. When comparing experiences of proportional sedation and continuous sedation, family members reported good relief with no differences in the relief of patient distress between the two sedation protocol [48]. Family members also considered that all possible measures had been taken to manage symptoms prior to the initiation of palliative sedation [52–54]. In these situations, palliative sedation was also perceived as providing effective relief, and family members saw no alternative means of achieving this. Monitoring equipment (BIS-monitor), was perceived as a means of verifying that the patient was comfortable and free from suffering [56]. This helped alleviate doubts the family members expressed regarding their own ability to assess the patient's symptoms.

## Discussion

The aim of this integrative literature review was to explore how palliative sedation of seriously ill individuals was experienced by their family members. The articles originated from Belgium, Israel, Japan, the Netherlands, Switzerland, the UK, and Taiwan. These are countries with different cultures but, at the same time, a high level of integration of palliative care, as described by Clark et al. [14].

Our findings demonstrate that having a relative who is undergoing palliative sedation is a significant source of emotional strain for family members, with negative implications for their health and well-being. This is clearly reflected in the categories. While palliative sedation was shown to *Relieve suffering* for the patient, and to some extent, also for the family members through the reassurance that the patient was no longer suffering from refractory symptoms, it simultaneously *Creates anxiety*, distress, and emotional suffering for the family. Many family members found it difficult to be certain that the patient was not suffering or feared that the patient might accidentally wake up. Similar findings have previously been reported [34, 35].

The strain experienced is also reflected in the category *Emphasizes importance of communication*. How well-informed family members felt varied between countries. In some countries, euphemisms such as “sleeping” or “being calm” were used instead of referring to sedation directly. The openness to talk about death and dying was also shown to differ between countries and cultures. This is in line with previous findings showing that while healthcare professionals in countries such as the Netherlands, Belgium, and Germany openly use the term palliative sedation in conversations with patients and families, it is often avoided in Spain [57]. In Korea, where discussions about prognosis and dying are commonly limited to protect family members from distress, palliative sedation may pose particular challenges [58]. In such contexts, family members may focus on maintaining hope rather than engaging in open discussions. These findings underscore the importance of culturally sensitive, honest, and clear communication to support understanding and emotional coping of palliative sedation. We believe that one reason for misunderstandings in communication may be the failure to name sedation and death explicitly. As healthcare professionals, we must be very clear when informing families about the type of palliative sedation being administered and what it entails.

Family members of both sedated and non-sedated patients rated their satisfaction with communication with healthcare professionals as equally satisfactory regardless of whether the patient had received palliative sedation or not. This may be interpreted as an indication that the healthcare staff succeeded in conveying the information effectively to both groups. However, the extent of the information provided and the need for information in the two groups was not stated. It is possible that the need for information was not perceived as equally urgent in the two groups. What did emerge from the findings, however, was that family members to sedated patients reported a better relationship with the healthcare staff. One possible explanation may be that family members were offered more time with the staff. Family members’ experiences of palliative sedation may also vary before, during, and after the sedation process. This aspect was beyond the scope of the present review but exploring temporal changes could be a valuable focus for future research.

The result showed that initiation of palliative sedation *Impacts time and timing* for family members. For them to feel at ease it was important that palliative sedation was initiated at the right time, before the patient’s symptoms became too severe. At the same time, family members experienced increased emotional strain the longer the sedation continued. There is notable global variation in the duration of palliative sedation between countries. Reported durations of sedation vary greatly, ranging from a few hours to several weeks [59]. Longer sedation times have been reported from Hong Kong [60] with durations ranging from 1 to 51,8 days while the study from Taiwan reported a median of 28.5 days [61]. In contrast, a Dutch study found that about half of patients under palliative sedation died within one day, and 46% within seven days [10]. The longer sedation durations observed in Hong Kong [60] may be explained by the continued oral intake for as long as possible, and the provision of enteral or subcutaneous fluids and other medical treatments for as long as deemed appropriate. Among those who received continuous deep palliative sedation in Netherlands, artificial nutrition and hydration were withheld in 91% of cases [4]. In Taiwan it was noted that palliative sedation is usually phased out near end-of-life, as patients tend to lose consciousness due to natural causes [61]. Following the termination of sedation, patients in the study died after 4.3 to 8.6 days. Continuous palliative sedation until death is generally considered most appropriate when associated with a shorter anticipated survival time, typically from several hours to a few days [59]. This appears to align with how palliative sedation is used in Sweden, more than half of the patients who received palliative sedation died within two days of its initiation, and only 4% received it for more than seven days [3]. Important questions about family members well-being, when patients are sedated for extended periods can be raised. The review found that prolonged sedation was shown to be distressing for family members. It is well known that being continuously present at the bedside during this time can be exhausting for family members, often involving sleep deprivation and ongoing uncertainty about when death will occur [34]. Although current guidelines do not specify a recommended duration for palliative sedation, sedation periods lasting up to or beyond one month appear inconsistent with recommendations that sedation should be reserved for the final phase of life [12]. We also believe that the early integration of palliative care is essential, as it allows time to establish a trusting relationship between healthcare professionals and family members, which is particularly important during palliative sedation.

Cultural beliefs and values, such as views on autonomy, dignity, the natural course of dying, and religious or spiritual interpretations of suffering, may influence family members’ perceptions of palliative sedation. As the patient’s condition deteriorates, the ability to communicate preferences may become limited [62]. In this situation, when palliative sedations is under consideration, questions of autonomy, shared decision-making, and the role of family members as proxies become increasingly central [12]. Once sedation has been initiated, the patient’s autonomy in the sense of decision-making may be reduced or lost. Family members therefore play a crucial role in supporting the patient and compensating for these limitations, thereby helping to preserve dignity and self-esteem [62, 63]. These complexities around autonomy and proxy decision-making can strongly influence how family members perceive and experience palliative sedation, particularly when communication is compromised. Cultural context may further shape expectations and experiences in these situations. For example, in Taiwanese society, where Buddhism is prevalent, a ‘good death’ is often associated with maintaining consciousness near the end of life, which may possibly explain reports that palliative sedation is sometimes phased out near end of life [61]. The articles included in the review predominantly reflect a European perspective, with a smaller number from Asia. Accordingly, this limited cultural diversity does not provide a sufficient basis to draw solid conclusions about the impact of cultural background. Future research exploring how cultural beliefs and values and religion shape family members’ experiences of palliative sedation would be of great interest.

The results also showed that family members experienced that palliative sedation *Affects the lifespan.* Some believed that it may shorten life and some expressed concern that it could be a form of euthanasia. These perceptions contrast with findings from another study which indicate that palliative sedation does not shorten lifespan but may prolong survival through improved symptom control and well-being [64]. In the UK, continuous deep sedation is less common than in Belgium and the Netherlands, and there appears to be greater concern among patients, family members and health care professionals that palliative sedation might hasten death [65]. Patients’ own wishes regarding palliative sedation are given significant consideration in Belgium and the Netherlands, [4], which may be partly attributed to the legalization of euthanasia in these countries, making the distinction between the two clearer. Palliative sedation may also be experienced as less emotionally burdensome than euthanasia as the primary intention is not to hasten death [65].

The results showed that family members could experience that palliative sedation *Provides a chance for farewell* and felt that it gave them sufficient time to say goodbye. It has also been described that some family members wish to delay the start of sedation in order to have more time for farewells [57]. When palliative sedation is planned, it signals that the patient’s life is nearing its end, which gives family members a timeframe to relate to and thus the opportunity to be present during the final stage. The opportunity to say farewell to loved ones have been described as conditions to experience death as good [38]. We see advantages in family members knowing that time is limited, as they frequently express a desire to understand how long the patient has left to live. When family members are aware that death is approaching, it is often easier for them to find the strength to remain by the patient’s side.

The results of this study revisit the important question about what is meant by the term palliative sedation. In the absence of international guidelines defining palliative sedation, it is difficult to determine how commonly it is used, how the term is understood across different countries and how these variations affect family members’ experiences. A review of 264 regional and national documents, including 13 guidelines from 10 countries, found that palliative sedation is recommended to relieve refractory symptoms [59]. Sedation was described in various ways; from light to deep, intermittent or continuous and terms like ‘calming’ or ‘relieving suffering’ were sometimes used without mentioning ‘sedation’. This creates ambiguity even in official guidelines. This lack of consistent terminology makes a shared understanding difficult, and the concept of palliative sedation may thus vary widely.

The lack of a clear and consistent definition of palliative sedation may also influence how family members experience and understand the practice. This raises an important question: Are we providing family members with sufficiently clear and transparent information about what palliative sedation involves? Simultaneously, expectations from both patients and family members regarding the alleviation of suffering continue to increase [4].

The level of palliative care integration within a country influences access to palliative sedation. In countries with a high level of palliative care integration, there is broad access to medications commonly used in palliative care and the option to receive palliative sedation, whereas in countries with lower levels of integration, such access may be limited or absent [14]. According to WHPCA, palliative care should be accessible to all individuals, regardless of diagnosis, age, or economic status [6]. Similarly, the UN Development Programme and SDG 10.2 and 10.3 emphasize the importance of equity, advocating equal rights and opportunities irrespective of gender, age, origin, or religion [39]. Globally, well-developed palliative care is available to only approximately 14% of the population and is predominantly accessible in Europe [1]. Access to palliative care remains uneven across Western European countries, despite recent positive developments [66]. In Sweden for example, where palliative care is generally well implemented, it is estimated that about half of dying patients do not receive specialized palliative care despite having clear needs. Moreover, only about one-third of all cancer patients worldwide have access to palliative care [67]. By 2060, the global need for palliative care is projected to increase by up to 87%. While palliative care is continuously improving and becoming more accessible, conditions still vary considerably both globally and within Europe [66]. The United Nations (UN) emphasises the need for increased funding to developing countries to train, recruit, and retain healthcare professionals in palliative care [39] in line with principles of equity and equal rights regardless of religion, age, gender, ethnicity, or disability. Yet substantial challenges remain for palliative care, extending beyond issues related to palliative sedation. We consider it essential that family members receive adequate support and information before, during, and after the palliative sedation of a relative, in order to maintain their own health and well-being during this particularly challenging time, in accordance with SDG 3 [39]. Without broader global implementation of palliative care, equitable access to palliative sedation cannot be achieved.

### Strengths and limitations

This integrative review included both qualitative and quantitative research which is a strength not least within an area such as palliative sedation where research is rather limited. It is nevertheless challenging to analyse and merge results from studies with differing methods to a comprehensive whole. While not limiting our searches to any specific parts of the world, only research articles in English language were included which can be viewed as a limitation since potential research in other languages thus were excluded.

The concept palliative sedation is rather wide and undefined and can include all from mild intermittent sedation to deep continuous sedation. The included articles covered both continuous and intermittent sedation, ranging from light to deep sedation, as well as proportional sedation, but foremost continuous deep sedation. In four of the articles, however, the type of sedation was not specified, and no articles were excluded according to type of palliative sedation. There is accordingly a limited possibility to draw conclusions about palliative sedation as one coherent phenomenon, however, this seem rather to be attributed to a lack of articles covering mild and intermittent palliative sedation, than to limitations within the study or the method used.

Palliative sedation is a relatively new treatment that has come into use in countries where palliative care has been implemented and reached at least some level of integration in care. The treatment has been developed and more established during more recent years and the search strategy thus focused on research published from 2005 after where this development was more marked. Including more early articles might risk including articles from where palliative sedation was something a bit different.

To ensure credibility and trustworthiness, we have aimed for transparency by providing a detailed description of the method used. By thoroughly following the steps outlined by Whittemore and Knafl [40], and carefully adhering to our predefined aim, as well as inclusion and exclusion criteria, the rigor of the results is strengthened. One article included in the results involved both caregivers and family members; however, it was retained because the results explicitly distinguished findings derived from caregivers from those derived from family members. The categories identified showed consistent patterns across the included studies. From a global perspective, both palliative care and, by extension, palliative sedation are unevenly distributed. While the review includes articles from a variety of countries, all represent settings where palliative care is relatively well integrated. We believe the findings may be transferable to other contexts with similarly high levels of palliative care integration and adequate access to opioids.

## Conclusion

Palliative sedation is a significant source of emotional strain for family members, despite type of sedation. The well-being of family members was affected while the patients received palliative sedation, and they felt worse the longer the sedation lasted. The information from the healthcare staff was of great importance and it was important that it was consistent. The study clearly demonstrates that family members have a significant need for support, regardless of the type of sedation used.

The absence of clear international guidelines for palliative sedation contributes to persistent conceptual ambiguity and wide variation in its meaning and use across countries. The lack of global registries further hampers systematic research and comparability of practices. Despite these challenges, palliative sedation remains a crucial component of palliative care for relieving refractory symptoms and alleviating family members’ anxiety about patient suffering. Greater conceptual clarity and international coordination are therefore essential to ensure that palliative sedation is understood, applied, and evaluated in a way that truly serves patients and their families at the end of life.

## Supplementary Information

Supplementary Material 1.

Supplementary Material 2.

## Data Availability

The datasets for this study are publicly available in the manuscript.

## Abbreviations

ALS: Amyotrophic lateral sclerosis
APA Psycinfo: American Psychological Association
CDS: Continuous deep sedation
CINAHL: Cumulative index of nursing and allied health
EAPC: European Association for Palliative Care
IAHPC: International Association for Hospice and Palliative Care
NICE: National Institute for Health and Care Excellence (quality assessment tools)
PEO: Population, Exposure, Outcome framework
PRISMA: Preferred Reporting Items for Systematic Reviews and Meta-Analyses
PubMed: Public/Publishers Medline
SDG: Sustainable development Goals
UK: United Kingdom
UN: United Nations
WHO: World Health Organisation
WHPCA: Worldwide Hospice Palliative Care Alliance
