# Supplementary material for: Family members’ experiences of palliative sedation -a systematic integrative review
Source: BMC Palliat Care. 2026 Feb 19;25:56. doi: 10.1186/s12904-026-02025-z (PMC12930745; doi:10.1186/s12904-026-02025-z)
Supplement: Supplementary file 1 — Supplementary Material 1. [file 12904_2026_2025_MOESM1_ESM.docx]

# Supplementary file 1

Supplementary file, table 1 Search strategy

| **Term** | **Keywords** |
| --- | --- |
| Palliative sedation | Text words in title or abstract: continuous sedation, deep sedation, palliative sedation, terminal sedation |
| Family members experience | Text words in title or abstract: experience*, attitude*, view*, feeling*, perspective*, ”famil* attitude*”,”life experience*” |
| Relationship | Text words in title or abstract:, famil*, ”loved one”, ”next of kin”, partner*, relative*, spouse, ”significant other*” |
| Limitations | Peer review  Publication year 2005-2025  Narrow by language: English  Over 18 years of age |
